# Multiple bronchial carcinoids associated with Cowden syndrome

**DOI:** 10.1007/s12020-024-03693-8

**Published:** 2024-02-14

**Authors:** Zsófia Tömböl, Judit Tőke, Géza Tóth, Zsolt Varga, Eszter Balázs, Erika Tóth, Lajos Gergely, Ľudovít Danihel, Márta Medvecz, Katalin Borka, Miklós Tóth

**Affiliations:** 1 Department of Medicine and Oncology, ENETS Center of Excellence, Faculty of Medicine, Semmelweis University, Budapest, Hungary; 2 Division of Endocrinology, 2nd Department of Medicine, Health Center, Hungarian Defense Forces, Budapest, Hungary; 3 Department of Endocrinology, Szent Lázár County Hospital, Salgótarján, Hungary; 4 Medical Imaging Centre, Department of Nuclear Medicine, Semmelweis University, Budapest, Hungary; 5 Medical Imaging Centre, Department of Radiology, Semmelweis University, Budapest, Hungary; 6 National Institute of Oncology, Department of Surgical and Molecular Pathology, Tumour Pathology Center, Budapest, Hungary; 7 Institute of Medical Biology, Genetics and Clinical Genetics, Faculty of Medicine, Comenius University in Bratislava, University Hospital Bratislava, Bratislava, Slovak Republic; 8 Institute of Pathological Anatomy, Faculty of Medicine, Comenius University in Bratislava, Bratislava, Slovak Republic; 9 Department of Dermatology, Venereology and Dermatooncology, ERN-Skin HCP, Faculty of Medicine, Semmelweis University, Budapest, Hungary; 10 Department of Pathology, Forensic and Insurance Medicine, Faculty of Medicine, Semmelweis University, Budapest, Hungary

**Keywords:** Multiple pulmonary carcinoids, Cowden syndrome, *PTEN* mutation

## Abstract

Cowden syndrome (CS) is a rare genetic condition due to the various germline mutations in the phosphatase and tensin homologue on chromosome ten (*PTEN*) tumour suppressor gene. As a result, CS is characterised by an increased risk of developing various benign and malignant tumours, such as thyroid, breast, endometrial and urogenital neoplasms, as well as gastrointestinal tract tumours. However, the neuroendocrine tumour association with CS is not elucidated yet. We present a case of a 46-year-old male patient diagnosed with testicular seminoma and follicular thyroid cancer in his medical history. Our patient met the clinical diagnostic criteria of Cowden syndrome. Genetic analysis established the clinical diagnosis; a known heterozygous *PTEN* mutation was detected [*PTEN* (LRG_311t1)c.388 C > T (p.Arg130Ter)]. Incidentally, he was also seen with multiple pulmonary lesions during his oncological follow-up. A video-assisted thoracoscopic left lingula wedge resection and later resections from the right lung were performed. Histological findings revealed typical pulmonary carcinoid tumours and smaller tumorlets. Somatostatin receptor SPECT-CT, ^18^F-FDG-PET-CT and ^18^F-FDOPA-PET-CT scans and endoscopy procedures could not identify any primary tumours in other locations. Our patient is the first published case of Cowden syndrome, associated with multifocal pulmonary carcinoids. Besides multiple endocrine neoplasia type 1, we propose Cowden syndrome as another hereditary condition predisposing to multiple pulmonary tumorlets and carcinoid tumours.

## Introduction

Cowden syndrome (CS) is a rare, autosomal dominantly inherited condition, most frequently caused by mutations in the phosphatase and tensin homologue on chromosome ten (*PTEN*) tumour suppressor gene located on 10q22-23 [1]. The prevalence of CS is approximately 1:200000-1:250000; however, this seems to be an underestimation explained by obvious diagnostic difficulties [1]. This phenotypically variable disorder is considered part of the PTEN hamartoma tumour syndrome spectrum disease, which also involves other genetic entities due to *PTEN* mutations such as Bannayan-Riley-Ruvalcaba syndrome, Proteus- and Proteus-like syndromes. Inactivating loss-of-function mutations of the *PTEN* tumour suppressor gene trigger the over-activation of the mammalian target of rapamycin (mTOR) pathway, which leads to increased cell proliferation, angiogenesis and reduced apoptosis [2].

Consequently, patients with CS have an increased risk of developing several benign and malignant tumours, most likely in adulthood, mainly follicular thyroid, breast, endometrial and renal cell neoplasms [1, 3]. Besides these cancers, other clinical characteristics are involved in the minor and major diagnostic criteria system of CS, such as macrocephaly, mental retardation, lipomas, trichilemmomas, fibromas, papillomatous skin lesions and hamartomatous polyps of the gastrointestinal tract [1, 3].

Despite the frequent occurrence of diverse tumours in patients with *PTEN* mutations, only a few cases of neuroendocrine tumours and gonadal germ cell tumours (6 compiled by Kouziki 2021; Tullius 2019, Pena-Cuoso 2022) have previously been reported in patients with CS [4–10].

Here, we present a case of Cowden syndrome associated with several pathognomic features and previously unreported multifocal pulmonary carcinoid.

## Case presentation

The 46-year-old male patient was referred to our endocrine oncology unit suspected of familial cancer syndrome in 2021. His mother had breast cancer, and several paternal relatives had multiple skin lesions; otherwise, we could not obtain a more detailed and medically documented family history. He has not got children. The slightly overweight patient (104.5 kg, 189 cm) had an increased head circumference (62 cm, >97 percentile), consistent with macrocephaly.

In 2000, at the age of 25 years, he was diagnosed with a left testicular tumour and underwent a left orchidectomy. The histological diagnosis revealed a purely seminomatous testicular tumour (pT1apN0cM0) without capsular infiltration and vascular invasion.

In 2016, at the age of 41 years, bilateral hypodense thyroid nodules (left lobe 3.8 cm; right lobe 2.8 × 1.7 cm) surrounded with hyperdense thyroid tissue were detected via computer tomography (CT) of the neck. Total thyroidectomy was performed, and the histological analysis revealed a poorly differentiated follicular thyroid cancer with capsular and vascular invasion (pT3pN0cM0) in the left lobe. The surrounding thyroid tissue showed follicular nodular thyroid disease. The tumour was classified as an ATA high-risk cancer. Beside suppressive levothyroxine treatment, our patient received two cycles of adjuvant radioiodine therapy (2854 and 3689 MBq) in 2016. From 2021, a continuous, slow increase of serum thyroglobulin was detected without any structural evidence of recurrence. Three more cycles of radioiodine treatment (3620, 2720 and 3590 MBq) were administered between 2021 and 2023. Post-therapeutic whole-body 131-iodine scans were repeatedly negative.

**Fig. 1:**
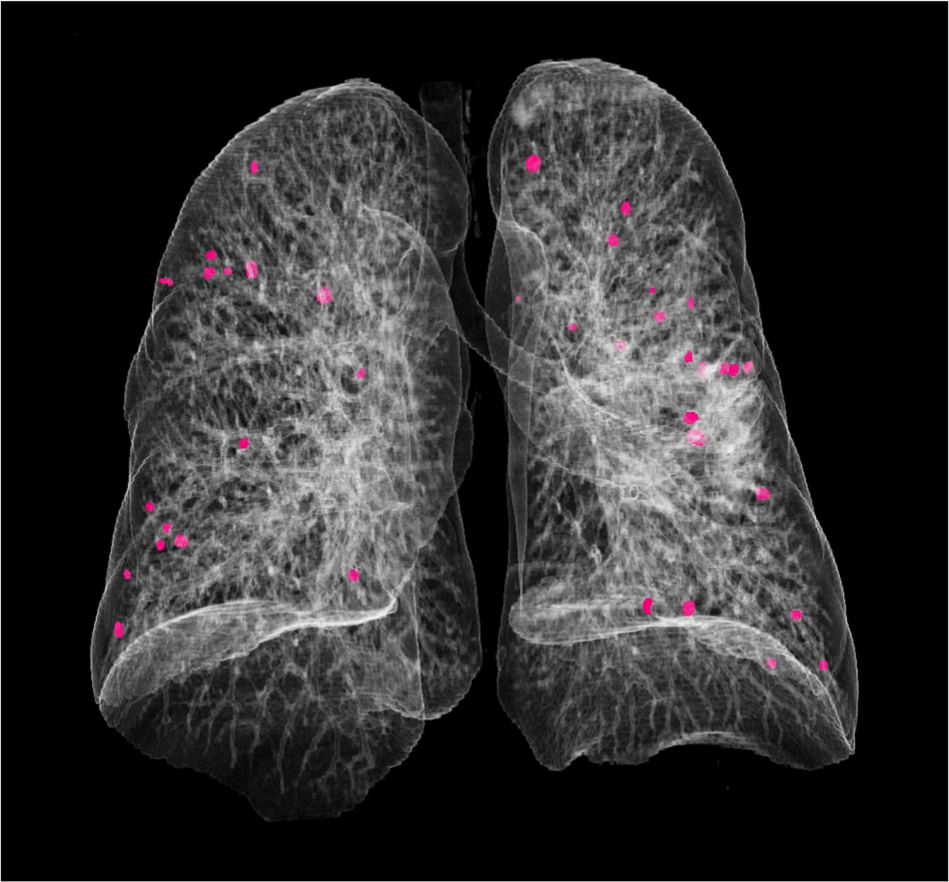
Nodule visualisation by computed tomography volume rendering. A three-dimensional (3D) volume rendering image from our patient demonstrates numerous pulmonary nodules (in magenta) with diameters between 3 and 12 mm (lung CT, 2022)

**Fig. 2:**
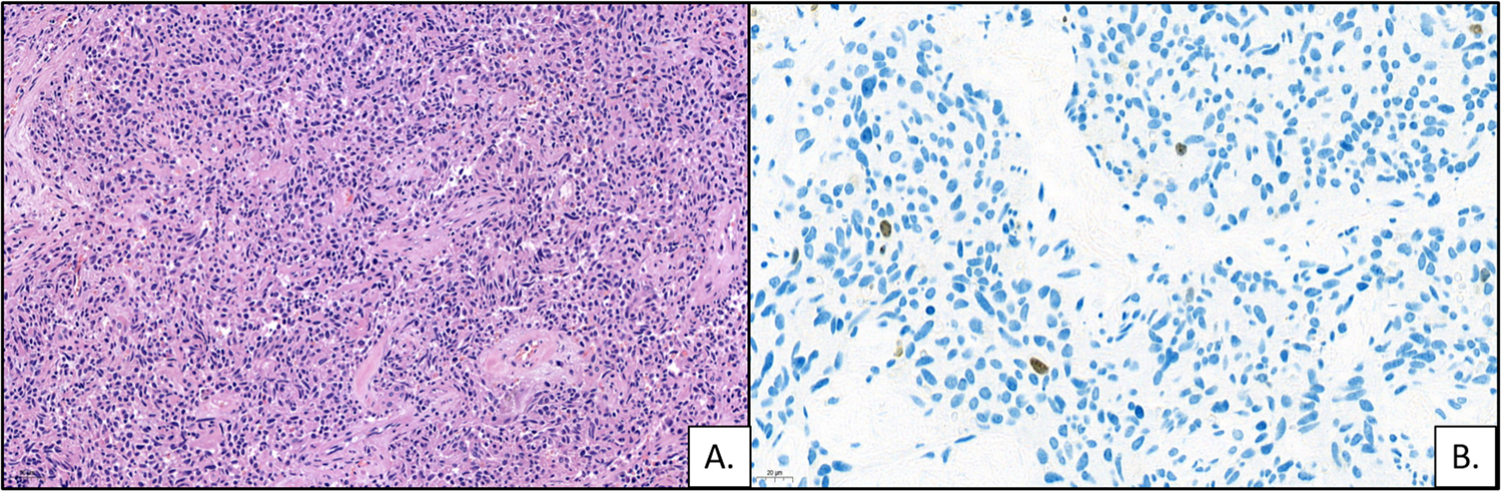
Photomicrographs of bronchial carcinoid. **A** Monomorphic neuroendocrine tumour cells with round to oval nuclei. No mitoses and necrosis are identifiable (H&E). **B** Immunostaining for Ki-67 shows a low proliferation rate (<1%)

Since 2016, as a result of thyroid cancer work-up, multiple bilateral pulmonary nodules with diameters 3–12 mm were detected. Nodule visualisation using volume-rendered computed tomography showed numerous pulmonary nodules on both sides and each lobe (Fig. 1). Furthermore, a 13x12x3 cm lipoma in the right chest wall, a 2 × 2 cm lipoma of the right thigh, as well as hemangiomas in the 8th and 12th thoracic vertebra and a renal cyst were identified. However, further diagnostic investigations, including ^18^F-FDG-PET-CT and ^18^F-FDOPA-PET-CT scans, were inconclusive. Therefore, to establish a definitive histological diagnosis, three pulmonary nodules (left S4-S5, right S6 and S10) were removed by wedge resections using video-assisted thoracoscopic surgery. Light microscopy revealed typical carcinoids without signs of necrosis, vascular, lymphatic or perineural invasion in each surgical specimen. The mitotic rate was between 0 and 1/10 high power fields. Immunohistochemistry was positive with chromogranin A and synaptophysin antibodies; the Ki-67 index was <1%, compatible with well-differentiated neuroendocrine tumours (Grade I) (Fig. 2). Immunohistochemistry for somatostatin receptor type 2 was negative. PTEN immunohistochemistry performed on the thyroid tumour and multiple pulmonary NETs proved to be negative.

**Fig. 3:**
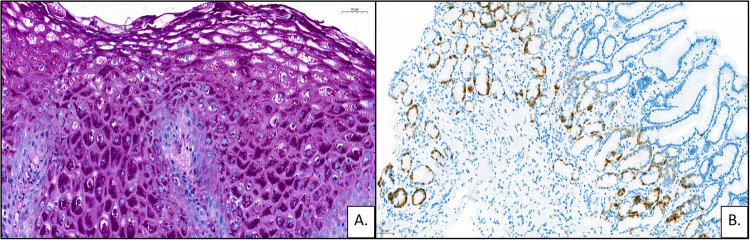
Upper endoscopic histological findings. **A** The oesophagus showed squamous cell hyperplasia with intracellular glycogen accumulation (PAS staining). **B** Linear enterochromaffin-like cell hyperplasia in the antral region (chromogranin-A immunostaining)

Upper and lower endoscopies were performed to exclude metastatic dissemination from a gastrointestinal primary, revealing hundreds of polypoid lesions throughout the gastrointestinal tract without histological signs of malignancy. Oesophageal biopsy showed squamous cell hyperplasia with intracellular glycogen accumulation (Fig. 3A). A gastric biopsy obtained from the antrum revealed linear enterochromaffin-like cell hyperplasia (Fig. 3B). A small polypoid lesion removed from the caecum was histologically verified as a hamartomatous polyp. Neuroendocrine tumourous lesion were not found anywhere. Somatostatin receptor scintigraphy with SPECT-CT was negatíve. Serum chromogranin A and 5-hydroxy indole acetic acid (5-HIAA) urinary excretion were normal. Serial thoracic-abdominal-pelvic follow-up CTs, performed until 2022, showed stable, multiple bilateral pulmonary nodules, each under 1 cm diameter.

On brain MRI, minor anomalies such as wider perivascular spaces, small aspecific foci in the cerebral white matter and contrast-enhanced developmental venous anomalies, each compatible with Cowden’s syndrome, were recognized. Cerebellar dysplastic gangliocytoma was not demonstrated.

Dermatological history included histologically verified scrotal papilloma, facial dermatofibroma, and oral fibroma. In addition, the dermatological examination performed in 2022 revealed multiple subcutaneous lipomas, cutaneous fibromas and hemangiomas, acral keratoses, macular pigmentation of the glans penis and oral and lingual papillomas.

Based on the medical history and clinical findings, a hereditary tumour syndrome was supposed. With four major (follicular thyroid cancer, macrocephaly, macular pigmentation of the glans penis and mucocutaneous lesions) and three minor criteria (lipomas, GI hamartoma, oesophageal glycogenic acanthosis), our patient met the clinical diagnostic criteria of Cowden syndrome. (Table 1) [3, 11] Genetic analysis of exons 3–9 of the *PTEN* gene using DNA obtained from peripheral leucocytes revealed a known heterogenous nonsense mutation in exon 5 [*PTEN* (LRG_311t1)c.388 C > T (p.Arg130Ter)]. Sanger sequencing of *PTEN* exon 5 of two formalin-fixed, paraffin-embedded pulmonary tumour samples was not informative because of poor DNA quality.

**Table 1:** Presence of the diagnostic criteria of Cowden syndrome in our patient according to National Comprehensive Cancer Network® (NCCN®) Clinical Practice Guidelines in Oncology version 3. 2023 [13]

**Major criteria**	Breast cancer
	Endometrial cancer
	**Follicular thyroid cancer**
	Multiple gastrointestinal hamartomas or ganglioneuromas
	**Macrocephaly (≥97 percentile: 58** **cm for females, 60** **cm for males)**
	**Macular pigmentation of the glans penis**
	**Mucocutaneous lesions:**
	One biopsy-proven trichilemmoma
	** Multiple palmoplantar keratosis**
	** Multifocal or extensive oral mucosal papillomatosis**
	Multiple cutaneous facial papules (often verrucous)
**Minor criteria**	Autism spectrum disorder
	Colon cancer
	**Oesophageal glycogenic acanthosis (≥3)**
	**Lipomas**
	Intellectual disability (i.e., IQ ≤ 75)
	Renal cell carcinoma
	Testicular lipomatosis
	Thyroid cancer (papillary or follicular variant of papillary)
	Thyroid structural lesions (e.g., adenoma, nodule(s), goiter)
	**Single GI hamartoma** or ganglioneuroma
	Vascular anomalies (including multiple intracranial developmental venous anomalies)

Criteria found in our patient are highlighted in bold text.

## Discussion

This unique patient with an unambiguous clinical and genetic diagnosis of Cowden syndrome developed several benign (gastrointestinal hamartomatous polyps, multiple lipomas) and malignant tumours (follicular thyroid cancer) widely accepted as part of Cowden syndrome. With four major and three minor criteria, the clinical diagnosis of CS was established. Furthermore, the genetic analysis confirmed the clinical diagnosis by detecting a known pathogenic nonsense mutation (c.388 C > T) of the *PTEN* tumour suppressor gene. This mutation results in a stop codon (p.Arg130Ter) and incomplete protein product. This single nucleotide variant of the *PTEN* tumour suppressor gene has been previously published and recorded several times in ClinVar Database as a pathogenic nucleotide variant associated with CS and PTEN hamartoma tumour syndrome [12].

Besides these tumours, our patient also developed two other malignancies, a testicular seminoma and multifocal pulmonary carcinoids. However, in the clinical diagnostic criterion system, neither of these tumours is considered an accepted constituent of Cowden syndrome [1, 3, 11, 13].

One of the first well-documented NET associated with Cowden syndrome was published in 2015 by Neychew et al. These authors suggested that their case was not only a coincidence of two rare entities, but NETs can be associated as new clinical features with CS [7]. Recently, Greidinger et al. published a new case and found 11 additional cases of NETs with retrospective analysis in patients diagnosed with CS. Considering the prevalences of CS and NETs in the United States, the relative risk of NETs associated with CS was estimated to have a sixfold increase compared to the general population, which raised the possibility that NETs should be involved in the diagnostic criteria of CS [4].

Pulmonary NETs maintain a spectrum of diseases involving diffuse or localised neuroendocrine cell proliferation and neoplasms [14]. Pulmonary NETs constitute a histologically heterogeneous group, which can be categorised due to their mitotic rate and the presence of necrosis from well-differentiated typical (low-grade) and atypical (intermediate-grade) pulmonary carcinoids to high-grade, poorly differentiated large-cell neuroendocrine and small-cell carcinomas, as well [15–17]. Neuroendocrine pulmonary tumorlets are, by definition, less than 5 mm [17]. In this sense, several neuroendocrine tumours in our patients represent tumorlets. The vast majority of pulmonary carcinoids are sporadic and solitary. Until now, the only known condition predisposing to pulmonary carcinoids is multiple endocrine neoplasia type 1. The prevalence of MEN1-pulmonary NET is 6.7% when only histology-verified cases and 30% when small bronchopulmonary nodules on CT are included [18, 19]. MEN1-associated NETs are multifocal [18].

Our patient presented is the fourth published case of Cowden syndrome associated with pulmonary NET. Langer et al. reported two patients; the first had two typical carcinoids, while the second had a unifocal atypical carcinoid [6]. The third case, published in Japan, had a unifocal atypical carcinoid [9]. Therefore, our patient seems unique since the obvious multifocality suggests an association with the established genetic disorder instead of a simple by-chance coincidence.

Our efforts to prove a causal relationship between pulmonary NETs and the *PTEN* genetic alteration were unsuccessful, because of poor DNA quality of paraffin-embedded tumoral tissue.

Until nowadays, seven cases of gonadal germ-cell tumours associated with Cowden syndrome were reported, three in male and four in female patients [5, 10]. Three of the seven gonadal tumours manifested in childhood or young adulthood. Interestingly, the gonadal tumours associated with Cowden syndrome represent different histological types of germ cell tumours (seminoma, mixed germ-cell tumour, mature and immature teratomas) [5].

At present, our patient is entirely asymptomatic; his thyroid cancer is well-controlled with periodic radioiodine treatment. Since his pulmonary carcinoid tumours lack somatostatin receptor type 2 expression, first-generation somatostatin analogue administration does not seem to be indicated. Considering the presently non-progressive nature of the pulmonary neuroendocrine tumours, we follow a watchful waiting strategy with 6 to 12 months of follow-up imaging, as suggested by our multidisciplinary team.

## Conclusion

Our observations with this unique case of Cowden syndrome might indicate the need to expand the PTEN-related tumour spectrum with multifocal neuroendocrine pulmonary tumours and probably also gonadal germ cell cancers. Establishing the diagnosis of Cowden syndrome significantly changes these patients’ diagnostic and surveillance strategies.
